# Supplementary material for: Weight Loss and Short-Chain Fatty Acids Reduce Systemic Inflammation in Monocytes and Adipose Tissue Macrophages from Obese Subjects
Source: Nutrients. 2022 Feb 11;14(4):765. doi: 10.3390/nu14040765 (PMC8878364; doi:10.3390/nu14040765)
Supplement: Supplementary file 1 [file nutrients-14-00765-s001.zip › nutrients-1555888-supplementary.pdf]

Table S1. Multiple linear regression model. Multiple linear regression model. mRNA gene expression of monocytes following propionate treatment.

Dependent Variable.	Independent Variable	Model				
		Coefficient	*p*	R^2	Adjusted R^2	*p*
**TNF-α** **F (2,20)**	FFAR3	0.256	0.094			
	HDAC2	0.863	0.015	0.41	0.35	0.005
**IL-6** F (2,20)	FFAR2	0.37	0.022			
	FFAR3	0.31	0.027	0.37	0.30	0.013
**NFKB1**	HDAC1	0.494	<0.001			
F (2,20)	HDAC2	0.307	0.254			
	HDAC9	0.382	0.004	0.83	0.80	<0.001
**RELA**	HDAC1	0.637	<0.001			
F (2,20)	HDAC2	0.301	0.182			
	HDAC9	0.339	0.003	0.90	0.88	<0.001
**MAPK1**	HDAC1	0.041	0.611			
F (2,20)	HDAC2	0.801	0.004			
	HDAC9	0.091	0.450	0.58	0.51	<0.001

TNF-*α*; tumour necrosis factor-*α*. IL-6; interleukin 6. [1] TNF-*α* measured in supernatant of LPS-stimulated monocytes. [2] mRNA gene expression measured in $2^{-\Delta CT}$.

Table S2. Multiple linear regression model. mRNA gene expression of monocytes following butyrate treatment.

	Independent Variable	Model				
		Coefficient	*p*	R^2	Adjusted R^2	*p*
**NFKB1** **F (2,20)**	FFAR2	0.625	0.020			
	FFAR3	-0.206	0.334	0.24	0.17	0.063
**NFKB1**	HDAC1	0.567	<0.001			
**F (2,20)**	HDAC2	0.313	0.098			
	HDAC9	0.097	0.456	0.82	0.79	<0.001
**RELA**	HDAC1	0.557	<0.001			
F (2,20)	HDAC2	0.309	0.033			
	HDAC9	0.171	0.090	0.89	0.87	<0.001
**MAPK1**	HDAC1	0.074	0.243			
F (2,20)	HDAC2	0.694	<0.001			
	HDAC9	0.242	0.006	0.87	0.85	<0.001

[1] mRNA gene expression measured in $2^{-\Delta CT}$.
